# Do Neuroscience Journals Accept Replications? A Survey of Literature

**DOI:** 10.3389/fnhum.2017.00468

**Published:** 2017-09-20

**Authors:** Andy W. K. Yeung

**Affiliations:** Oral and Maxillofacial Radiology, Applied Oral Sciences, Faculty of Dentistry, The University of Hong Kong Hong Kong, Hong Kong

**Keywords:** journal editorial practices, information science, literature-based discovery, neuroscience, replication

## Abstract

**Background**: Recent reports in neuroscience, especially those concerning brain-injury and neuroimaging, have revealed low reproducibility of results within the field and urged for more replication studies. However, it is unclear if the neuroscience journals welcome or discourage the submission of reports on replication studies. Therefore, the current study assessed the explicit position of neuroscience journals on replications.

**Methods**: A list of active neuroscience journals publishing in English was compiled from Scopus database. These journal websites were accessed to read their aims and scope and instructions to authors, and to assess if they: (1) explicitly stated that they accept replications; (2) did not state their position on replications; (3) implicitly discouraged replications by emphasizing on the novelty of the manuscripts; or (4) explicitly stated that they reject replications. For journals that explicitly stated they accept or reject replications, their subcategory within neuroscience and their 5-year impact factor were recorded. The distribution of neuroscience replication studies published was also recorded by searching and extracting data from Scopus.

**Results**: Of the 465 journals reviewed, 28 (6.0%) explicitly stated that they accept replications, 394 (84.7%) did not state their position on replications, 40 (8.6%) implicitly discouraged replications by emphasizing on the novelty of the manuscripts, and 3 (0.6%) explicitly stated that they reject replications. For the 28 journals that explicitly welcomed replications, three (10.7%) stated their position in the aims and scope, whereas 25 (89.3%) stated in within the detailed instructions to authors. The five-year impact factor (2015) of these journals ranged from 1.655 to 10.799, and nine of them (32.1%) did not receive a 5-year or annual impact factor in 2015. There was no significant difference in the proportions of journals explicitly welcomed replications (journals with vs. without impact factors, or high vs. low impact factors). All sub-categories of neuroscience had at least a journal that welcomed replications.

**Discussion**: The neuroscience journals that welcomed replications and published replications were reported. These pieces of information may provide descriptive information on the current journal practices regarding replication so the evidence-based recommendations to journal publishers can be made.

## Introduction

Replications, as defined by Schmidt (2009), can be broadly classified as direct replication, which is a “repetition of an experimental procedure”; and conceptual replication, which is a “repetition of a test of a hypothesis or a result of earlier research work with different methods”. Whereas direct replication provides an insight into the consistency of the presence or absence of an effect, conceptual replication can take a step further to generalize results to a larger or different population, verify the hypotheses of previous works and even evaluate data from new perspectives by improved methods such as experimental paradigm and data analysis (Schmidt, 2009; Evans, 2017).

Replications are particularly important for the neuroscience field, as the survey and analyses performed by Button et al. (2013) have concluded that neuroscience studies in general have small sample sizes with low statistical power, overestimation of effect size; and have low reproducibility. These findings were consistent with the analyses conducted by Poldrack et al. (2017) which simultaneously pointed out that the flexibility of analysis workflows, particularly those regarding neuroimaging data, may further reduce the reproducibility of results. A recent replication study which attempted to reproduce 17 correlational observations between brain structure and behavior reported that 30% of their replications resulted in effect sizes in an opposite direction to the original results, whereas most of the remaining replications reported a smaller effect size than those reported from the original report (Boekel et al., 2015).

Though multiple analysis articles recommended the publication of well-designed neuroscience replication studies (Button et al., 2013; Boekel et al., 2015; Evans, 2017; Poldrack et al., 2017), it is still unclear if neuroscience journals are explicitly supportive of publication of replications. A recent survey on psychology journals has found that only 3% of the surveyed journals have objectively stated they welcome submissions of replication studies (Martin and Clarke, 2017). This is highly relevant because 8% of neuroscience publications in 2015 were of psychology as assigned by Web of Science (Yeung et al., 2017b). A similar survey to neuroscience journals will benefit the scientific community by revealing if these journals heeded the call for acceptance of replications. Therefore, the main aim of the current study was to examine the information provided on the websites of neuroscience journals to determine if they welcome, discourage or reject replication studies. This would provide descriptive information on the current journal practices regarding replication so the evidence-based recommendations to journal publishers can be made. A secondary aim was to search the neuroscience literature over the last decade to assess if replication studies were published in those journals that explicitly welcome them.

## Materials and Methods

Neuroscience journals were identified by searching Scopus, a subscription based database hosted by Elsevier. There were 680 entries in the neuroscience category. After removing the inactive, the non-English and the non-journals, there were 465 journals. The complete list of these 465 journals was uploaded as Supplementary Data Sheet S1. The websites of these 465 journals were accessed to review their “aims and scope” (or equivalence, e.g., “about the journal”) and “instructions to authors” (or equivalence, e.g., “guide for authors”) sections. Following the classification by Martin and Clarke (2017), the journals were evaluated to see if they: (1) explicitly stated that they accept replications; (2) did not state their position on replications; (3) implicitly discouraged replications by emphasizing the novelty of the manuscripts; or (4) explicitly stated that they reject replications. For journals that explicitly stated they accept or reject replications, their sub-category within neuroscience as listed by Scopus was recorded, as well as their 5-year impact factor as listed in Journal Citation Reports released by Clarivate Analytics. This information may help researchers to identify target journals for their replication studies, as many institutions consider impact factor, though controversial, as an important metric that gauges the academic performance of their faculty (Abbott et al., 2010). Scopus was searched to identify publications with the phrase “replication study” in the title and published in journals classified as neuroscience journals by Scopus. The publication year and journal of them were recorded.

Chi-squared tests were performed to evaluate if there was a significant difference in the ratio of journals explicitly accepting replications between journals with and without impact factor, and between journals with high and low impact factor (by median split). Test results were statistically significant if *p* < 0.05. Statistical analyses were performed with SPSS 24.0 (IBM, New York, NY, USA).

## Results

**Figure 1:**
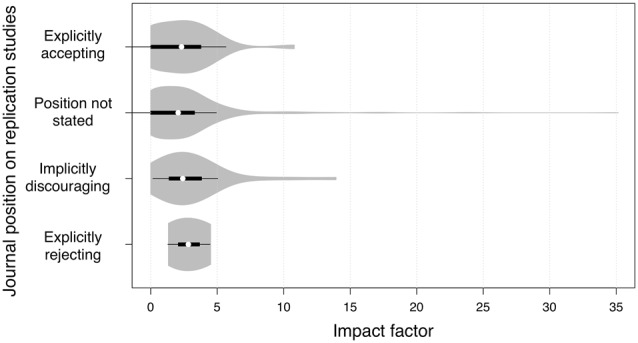
Violin plots illustrating the impact factors of journals with various positions on replication studies. White circles showed the medians; box limits indicated the 25th and 75th percentiles; whiskers extended 1.5 times the interquartile range from the 25th and 75th percentiles; and polygons represented density estimates of data and extended to extreme values. Journals that did not mention if they accept or reject replications had the widest range of impact factors, from 0 (no impact factor) to 35.142.

Of the 465 journals reviewed, 28 (6.0%) explicitly stated that they accept replications, 394 (84.7%) did not state their position on replications, 40 (8.6%) implicitly discouraged replications by emphasizing on the novelty of the manuscripts, and 3 (0.6%) explicitly stated that they reject replications. The median impact factors for journals in these position categories were 2.318, 2.066, 2.412 and 2.831, respectively (Figure 1). The percentages of journals without impact factor in these categories were 32.1%, 27.4%, 12.5% and 0%, respectively.

**Figure 2:**
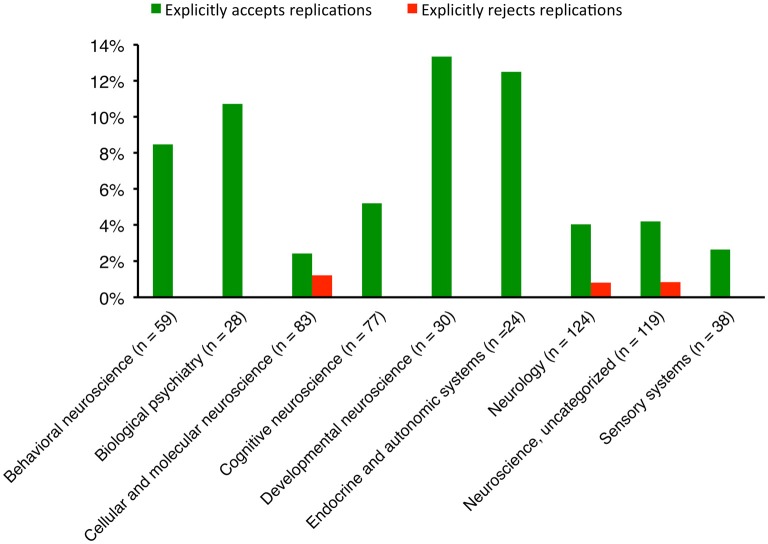
The proportions of neuroscience journals in subcategories that have an explicit position on replication studies. The subcategories, as assigned by Scopus, were not mutually exclusive.

For the 28 journals that explicitly welcomed replications, three (10.7%) stated their position in the aims and scope, whereas 25 (89.3%) stated it within the detailed instructions to authors (Table 1). No journal explicitly stated its position in both aims and scope and instructions to authors. The 5-year impact factor (2015) of these journals ranged from 1.655 to 10.799, and nine of them (32.1%) did not receive a 5-year or annual impact factor in 2015. All sub-categories of neuroscience had at least a journal that welcomed replications (Figure 2).

**Table 1:** Neuroscience journals with a clear position on replication studies.

Journal	Location of statement^a^	5-year IF (2015)^b^	Number of replications^c^
**Explicitly encourage replications**			
*Biological Psychiatry*	2	10.799	8
*Molecular Autism*	2	5.184	1
*Journal of Experimental Psychology *General*:*	2	5.105	2
*Multiple Sclerosis Journal*	2	4.546	1
*Molecular Brain*	2	4.159	0
*Progress in Neuro-Psychopharmacology and Biological Psychiatry*	2	4.111	1
*Hormones and Behavior*	2	3.923	0
*Journal of Research on Adolescence*	2	3.724	0
*Neural Development*	2	3.529	0
*Hormones and Cancer*	1	3.167*	0
*International Journal of Psychophysiology*	2	2.817	1
*Journal of Intellectual Disability Research*	2	2.734	0
*Therapeutic Advances in Neurological Disorders*	2	2.642*	0
*Brain and Behavior*	2	2.403	0
*Attention, Perception and Psychophysics*	2	2.233	0
*Focus on Autism and Other Developmental Disabilities*	2	2.036	0
*Journal of ECT*	2	1.770	0
*Human Factors*	2	1.767	
*Developmental Neurorehabilitation*	2	1.655	0
*Biological Psychiatry: Cognitive Neuroscience and Neuroimaging*	2	NA	0
*Brain Sciences*	2	NA	0
*Clinical Neurophysiology Practice*	1	NA	0
*ENeuro*	2	NA	0
*IBRO Reports*	1	NA	0
*Journal of Contextual Behavioral Science*	2	NA	0
*Thyroid Research*	2	NA	0
*Timing and Time Perception*	2	NA	0
*Translational Neurodegeneration*	2	NA	0
**Explicitly discourage replications**			
*Epilepsia*	2	4.507	1
*Neurogenetics*	1	2.831	2
*Brazilian Journal of Medical and Biological Research*	1	1.330	0

^a^1: Statement appeared in “About the Journal” or “Aims and Scope” Section. 2: Statement appeared in “Guide for Authors” or “Instructions for Contributors” Section. ^b^IF: impact factor. Asterisk (*) means the 5-year impact factor (2015) is not available for the journal and its 2015 impact factor is listed instead. NA means no impact factor is available for the journal. ^c^The number of replication studies indexed in Scopus.

There were 343 neuroscience journals with an impact factor, with 19 of them explicitly welcomed replications; there were 122 neuroscience journals without any impact factor, with nine of them explicitly welcomed replications. The ratio did not significantly differ between the groups (*χ*^2^ = 0.54, *p* = 0.46). The median impact factor of the 343 journals with an impact factor was 2.796. By median split, there were 11 and 8 journals explicitly welcomed replications in the journal groups with high and low impact factors respectively. The ratio did not significantly differ between the groups (*χ*^2^ = 0.43, *p* = 0.51).

For the three journals that explicitly rejected replications, two (66.7%) stated it in the aims and scope and one (33.3%) stated in the instructions to authors. Their 5-year impact factor (2015) ranged from 1.330 to 4.507.

**Figure 3:**
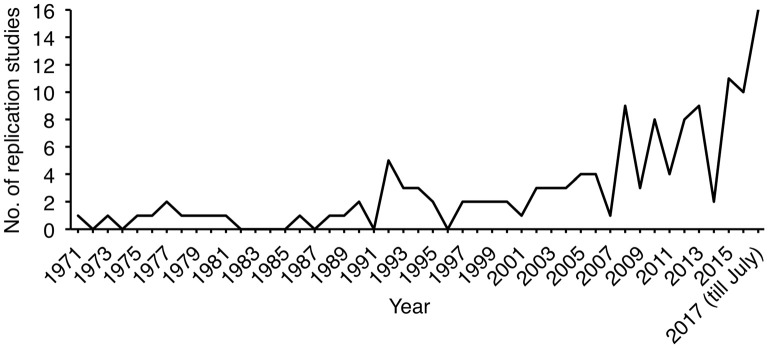
Line plot illustrating the annual publication count of replication studies in neuroscience.

The search for neuroscience replication studies indexed in Scopus resulted in 143 records, in which three were errata, one was a note (discussed the necessity of replication studies instead of an actual replication) and the other one article was indexed twice. After excluding these, 138 records remained, in which 130 were articles and eight were letters. These 138 publications were published in 73 journals. Most of them were lab-based instead of field-based, and focused on neurogenetics. Fourteen (10.1%) were published in journals that explicitly welcomed replications, 18 (13.0%) were in journals that implicitly discouraged replications, and 3 (2.2%) were in journals that explicitly rejected replications. At least one replication study was published annually since 1997 (Figure 3). Interestingly, apart from *Biological Psychiatry* that published eight of these (8/138 = 5.8%), other journals that explicitly accept replications did not seem to contribute much to these publications (Table 1). On the other hand, though *Neurogenetics* explicitly rejects replications by stating that “data merely confirming previously published findings are not acceptable”, two genetic studies were published in 2010 and 2012 to confirm previous findings of genetic associations.

Apart from *Biological Psychiatry*, four other journals published at least five replication studies, namely *Psychiatry Research* (*n* = 8), *Schizophrenia Research* (*n* = 7), *Journal of Affective Disorders* (*n* = 6) and *eLife* (*n* = 5). All of them, except *Biological Psychiatry*, did not state their position on replications.

When the subcategories of neuroscience journals were considered, biological psychiatry and neurology journals published 41 and 24 replication studies respectively, followed by uncategorized neuroscience journals (*n* = 22), cellular and molecular neuroscience (*n* = 16) and behavioral neuroscience (*n* = 10). Journals in other subcategories published fewer than 10 replications.

## Discussion

The main aim of this study was to evaluate if peer-reviewed neuroscience journals have stated their position on replication studies of previously published articles. The current study surveyed 465 neuroscience journals that are active, published in English and indexed in Scopus. It was found that 28 (6%) journals explicitly stated that they accepted replications. They had a broad range of impact factor, and one-third of them did not have an impact factor. Results have indicated that there was no significant difference in the proportion of journals explicitly welcomed replications between journals with and without impact factors, and between journals with high and low impact factors. Meanwhile, the results suggested that the median impact factor of each group of journals was comparable regardless of their position on replication studies. In terms of journals that explicitly accept replications, it seems neuroscience journals (6%) are more supportive than psychology journals (3%) in general (Martin and Clarke, 2017). Nonetheless, Martin and Clarke (2017) reported that 4.5% of the biological/neuropsychology branch of psychology journals accepted replications, which is more comparable to the current findings. Notwithstanding, it should be emphasized that all these figures indicated that most journals did not explicitly welcome replications. Whereas journals in biological psychiatry and neurology subcategories published 41 and 24 replication studies, respectively, it should be noted that the number of biological psychiatry journals (*n* = 28) was much smaller than the latter (*n* = 124). The replication studies published in biological psychiatry journals were mainly focused on genetic associations with neurological disorders.

Readers should be aware that the position of a journal on replication studies might be stated in isolated editorials, such as the cases for *Cortex* (Chambers, 2013) and *PLoS Biology* (Patterson and Cardon, 2005). Additionally, some journals might publish special issues devoted to replication studies, such as *Cognitive, Affective and Behavioral Neuroscience* (*CABN, Barch and Yarkoni, 2013*); or participate in reproducibility projects that aimed at reproducing selected studies, such as *eLife* (Errington et al., 2014). The instructions to authors from *Cortex* and *CABN*, however, actually stated that the submitted manuscripts “must report important and novel material” and “should provide a novel approach to a question (or set of questions) relevant to the mission of CABN, and/or provide new directions for empirical research” respectively. The implications of these pieces of information are threefold: first, the position of certain journals on replication studies may change over time; second, researchers might not easily notice these call for replications as they were not accessible from aims and scope or instructions to authors; and finally, the replications specifically called for might not match with the capacity or interest of the researchers.

The results from the current study showed that the majority of the neuroscience replication studies indexed in Scopus (89.9%) were published in journals that did not explicitly accept replications (94.0% of surveyed journals). This was noteworthy and encouraging as it implied that researchers could actually consider submitting replication studies to journals that did not state their positions. As Button et al. (2013) and Poldrack et al. (2017) urged for the provision of incentives to encourage replication studies, particularly of neuroimaging, the respective community has created free online databases containing functional neuroimaging data pooled across the globe, such as the OpenfMRI Project (Poldrack et al., 2013) and NeuroVault (Gorgolewski et al., 2015). A replication award has been established by the Organization for Human Brain Mapping to recognize the best replication study in neuroimaging^1^. It was reported that publications of the neuroscience and particularly the neuroimaging fields have been growing steadily (Yeung et al., 2017a,b,c), it could be intuitive to think that the number of published replication studies would also demonstrate a steady growth. However, Figure 3 has illustrated the annual publication count of replications fluctuated and never exceeded 20. This strongly suggests the importance of raising journals’ acceptance of replications as there were still very few replications published in recent years. It should be noted that the actual number of replication studies in neuroscience might be higher than what was reported in the current study, due to the search strategy and the fact that some replication studies may not label themselves in the title. It should also be noted that not all neuroscience journals and articles were indexed in Scopus, though it was reported that Scopus had broader journal coverage than Web of Science (Falagas et al., 2008).

Readers should read the replication studies together with the original studies to better interpret the results and conclusions. This highlights an inevitable issue: how should we label the replication studies? Guidelines exist for reporting different types of original research articles, such as STROBE for observational studies, CONSORT for randomized controlled trials and PRISMA for systematic reviews of intervention studies (Simera et al., 2008). There also exist guidelines for reporting different types of neuroscience research, particularly those concerning brain imaging (Poldrack et al., 2008; Ridgway et al., 2008). These guidelines offer recommended checklists of items to be reported in details for readers to follow and reproduce the studies. Replication studies should be labeled clearly in their title so that they are easily identified. Alternatively, it would be equally effective if the scientific community recognizes replication studies in the list of accepted forms for submissions. All these propositions require effort and consensus from all publishers to be implemented. It is recommended that all neuroscience journals clearly indicate their position on replications (welcome/discouragement) in their aims and scope or instructions to authors. It would be even better if all neuroscience journals clearly indicate that they encourage replications.

## Author Contributions

AWKY is responsible for all parts of the work.

## Conflict of Interest Statement

The author declares that the research was conducted in the absence of any commercial or financial relationships that could be construed as a potential conflict of interest.

## Footnotes

^1^https://www.humanbrainmapping.org/i4a/pages/index.cfm?pageID=3731

## Supplementary Material

Supplementary Data Sheet S1 List of neuroscience journals screened in the current study.
